# Xiaopiyishen Herbal Extract Granule Improves the Quality of Life among People with Fatigue-Predominant Subhealth and Liver-Qi Stagnation and Spleen-Qi Deficiency Syndrome

**DOI:** 10.1155/2012/509705

**Published:** 2012-07-18

**Authors:** Xiao-lin Xue, Xiu-yan Wu, Jian-min Xing, Li Li, Yan Zhao, Jia-jia Wang, Ya-jing Zhang, Qing-bo Wang, Yu Tang, Guan-ru Li, Ping Han, Zhen Li, Wen-ping Wang, Tian-fang Wang

**Affiliations:** ^1^Department of Diagnostics of Traditional Chinese Medicine, Preclinical School, Beijing University of Chinese Medicine, Beijing 100029, China; ^2^Center of Evidence-Based Medicine, Preclinical School, Beijing University of Chinese Medicine, Beijing 100029, China; ^3^Preventive Treatment and Health Management Center, The First Affiliated Hospital of Henan University of TCM, Zhengzhou 450000, China; ^4^Department of Acupuncture and Moxibustion, The First Affiliated Hospital of Henan University of TCM, Zhengzhou 450000, China; ^5^Department of Rehabilitation Medicine, The Affiliated Hospital of Liaoning University of TCM, Liaoning Province, Shenyang 110032, China; ^6^Health Examination Centre, Beijing Xiao Tang Shan Hospital, Beijing 102211, China; ^7^Department of Endocrinology, The First Affiliated Hospital of Henan University of TCM, Zhengzhou 450000, China; ^8^Clinical Trial Institution, The Affiliated Hospital of Liaoning University of TCM, Liaoning Province, Shenyang 110032, China

## Abstract

To observe the effects of Xiaopiyishen Herbal Extract Granule (XPYS-HEG) on the quality of life in people with fatigue-predominant subhealth (FPSH) and liver-qi stagnation and spleen-qi deficiency syndrome, the participants were allocated randomly to the treatment group (XPYS, *n* = 100) and the control group (placebo, *n* = 100) in this study. The study period was 18 weeks (6 weeks for the intervention and 12 weeks for followup). The results show that there were no differences between the two groups for the scores of eight factors on the SF-36 (Chinese version of the SF-36 universal quality-of-life scale) at baseline. Compared with the baseline score, intervention with XPYS-HEG led to a significant increase in scores for the factor of bodily pain at the end of the 6th week. Compared with the score at the end of the 6th week, the score for the factor of mental health in the XPYS group significantly increased at the end of the 18th week. Therefore, XPYS-HEG could partially improve the quality of life for people with FPSH and liver-qi stagnation and spleen-qi deficiency syndrome, which can ease bodily pain, stimulate a positive mood, and ease a negative mood.

## 1. Introduction

Fatigue is a common health-related complaint, a frequent complication of diseases, and the chief symptom of chronic fatigue syndrome (CFS) or fatigue-predominant subhealth (FPSH). Chronic fatigue syndrome is an illness characterized by disabling fatigue lasting at least 6 months, accompanied by several other symptoms [1, 2].

Subhealth (also referred to as suboptimal health) is a new concept that has been described by Chinese scholars [3]. Subhealth refers to a status between healthy and diseased states and is characterized by reduced vitality, function, and adaptive capacity lasting for at least 3 months. Subhealth is not considered a health condition and does not meet the clinical and subclinical diagnostic criteria of some diseases at this stage [4].

Various uncertain and abnormal physical and mental complaints presented by individuals with subhealth status have no conclusive laboratory markers. The main symptoms of subhealth include fatigue, sleeping disorders, amnesia, bodily pain, anxiety, and depression.

Subhealth has become a public health challenge in China because a surprisingly large number of Chinese people suffer with this condition. The prevalence of subhealth was found to be high among college and university staff according to a regional survey in China.The survey suggested that individuals with subhealth status were more likely to be women and middle aged. The most common risk factors were occupational stress, psychological factors, bad habits and behaviors, lack of relaxation and physical exercise, working extended hours, and air and noise pollution [5, 6].

People with subhealth report a poor quality of life. Therefore, it has become increasingly important to identify effective intervention methods. TCM is characterized by the concept of holism, which promotes the use of numerous intervention methods and plays an important role in the treatment of subhealth status. 

The condition of subhealth status maybe divided into various types according to the predominant symptoms. Fatigue-predominant subhealth is characterized by obvious fatigue and represents the most frequently reported type of subhealth status [7]. In our prior study [8, 9], we reported that liver-qi stagnation and spleen-qi deficiency syndrome is the most common TCM syndrome observed in people with FPSH. This syndrome is marked by hypochondriac pain, depression or irritation, abdominal distention, loose bowel, and lassitude. The life quality of people with FPSH is impaired; therefore, the improvement of the quality of life is an important goal of the TCM interventions investigated in our study [10].

In a previous investigation, the total score of the Fatigue Scale 14 was used to evaluate the efficacy and safety of Xiaopi Yishen herbal extract granule (XPYS-HEG) in the treatment of people with FPSH due to liver-qi stagnation and spleen-qi deficiency. The fatigue status and the grade of liver-qi stagnation and spleen-qi deficiency syndrome were also recorded. It was reported that XPYS-HEG relieved fatigue and other symptoms associated with liver-qi stagnation and spleen-qi deficiency syndrome [11].

The purpose of the paper was to analyze the effect of XPYS-HEG on the quality of life in people with FPSH and liver-qi stagnation and spleen-qi deficiency syndrome.

## 2. Methods

### 2.1. Study Protocol

A placebocontrolled multicenter clinical trial with a randomized, double-blinded, and parallel design was completed by 3 participating centers across mainland China (Beijing, Henan, Liaoning). Randomization schedules were generated by a statistician using the statements of PROC PLAN of SAS (version 9.1.3) and assigning equal numbers of patients to each of the groups. Block sizes of 2 and 4 were used to balance the assignments across groups and to prevent decoding of the system. Assignments were stratified within the centers. The allocations were placed in numbered opaque envelopes to be opened by the doctors in the presence of the participants. The process of disclosed blinding included two steps. The first disclosure was performed after completion of the reviewing of the data, that is, only disclosing the code A or B of each case to the statistician. The second was performed after completing the statistical analysis and report, that is, disclosing the corresponding group of A and B. The removal of the blinding during the process of this trial was executed by the researchers when serious adverse event occurred or the participant needed emergency medical treatment. This trial was intended to be terminated if all of the allocations were disclosed or if the proportion of patients for whom the blinding was removed exceeded 20%.

### 2.2. Participants

The sample size of 200 was estimated using the formulation of *n* = (*U*_*α*_+*U*_*β*_)^2^ × 2*P* × (1 − *P*)/(*P*_1_−*P*_0_)^2^ [12] with type I error 0.05 and type II error 0.1. The participants were recruited among patients receiving a physical check up and outpatients during the period of March 2008 and February 2009 at Beijing Xiao Tang Shan Hospital (80 cases), the First Affiliated Hospital of the Henan College of Chinese Medicine (60 cases) and the Affiliated Hospital of the Liaoning University of Chinese Medicine (60 cases).

### 2.3. Diagnostic Criteria

#### 2.3.1. Diagnostic Criteria for FPSH

According to the “Clinical Guidelines of Chinese Medicine on Subhealth,” the research group developed the following diagnostic criteria for FPSH [13]: (1) chief complaint: persistent or recurrent fatigue lasting more than 3 months; (2) exclusion: a disease that may lead to fatigue, with no obvious abnormalities detected through a routine physical examination; (3) total scores reaching 3 points or more on the Fatigue Scale 14 (FS-14) [14, 15]. The routine physical examinations include routine analyses of the blood, urine and stool, blood pressure, liver and kidney function, blood lipid profile, fasting blood sugar, abdominal B ultrasound, an ECG, and a chest X-ray.

#### 2.3.2. TCM Differentiation Standard

In accordance with the “criteria for the diagnosis and evaluation of the therapeutic effects of treatments of diseases and syndromes in traditional Chinese medicine,” “Differential diagnosis of the syndromes of Chinese medicine” released in 1995, which were issued by the State Administration of Traditional Chinese Medicine of the People's Republic of China, and “The guiding principles for the clinical study of new drugs for use in traditional Chinese medicine” released in 2002, combined with the characteristics of subhealth, the standards of liver-qi stagnation and spleen-qi deficiency syndrome in FPSH are formulated as follows: (1) chest or hypochondriac fullness, distending pain, or wandering pain; (2) low mood, irritability, or emotional instability; (3) reduced appetite, abdominal distension/relieved by pressure, loose stools, or diarrhea; (4) alternate loose and dry stools; (5) enlarged and tooth-marked tongue. Participants can be diagnosed with liver-qi stagnation and spleen-qi deficiency syndrome if they meet (1) or (2) and (3) or (4) or (5), excluding the apparent thermal effects, such as a red tongue with a yellow coating (refer to pulse, such as wiry pulse, moderate pulse, weak pulse, or feeble pulse).

### 2.4. Inclusion Criteria

The inclusion criteria for this study were as follows: (1) meet the diagnostic criteria described above for FPSH and liver-qi stagnation and spleen-qi deficiency syndrome according to the FPSH of TCM syndrome differentiation standards; (2) between 18 and 60 years of age; (3) education: junior high school and above; (4) no fatigue interventions (including antifatigue healthcare supplements) taken within the past month; (5) participants signed informed consent.

### 2.5. Exclusion Criteria

The exclusion criteria were as follows: (1) upper respiratory tract infection, trauma, acute medical history, and so forth. Within the past week (2) pregnant or lactating women and women planning a pregnancy within the next six months; (3) a history of mental illness or family history of psychiatric disorders.

### 2.6. Dropout Criteria

Participants were considered as dropouts when he/she did not complete the entire observation period. Participants who stopped taking the medication with less than one treatment cycle were not counted as dropouts.

### 2.7. Ethics and Consent

The study protocol conforms to the Helsinki Declaration [16] and the research regulations for Chinese clinical trials. The Ethics Committee of the Affiliated Dongzhimen Hospital of the Beijing University of Chinese Medicine reviewed and approved the study protocol. All participants were required to provide written informed consent before participation in the study.

### 2.8. Treatment

A total of 200 participants were randomly divided into the XPYS-HEG intervention group (XPYS, *n* = 100) and the placebo group (*n* = 100). The participants were given XPYS-HEG (10 g Radix Astragali, 2 g Radix Ginseng, 6 g Pericarpium Citri Reticulatae, 6 g Rhizoma Cyperi, 6 g Radix Angelicae, and 6 g Fructus Lycii) or placebo with same packaging as that used for XPYS-HEG. The participants took one bag half an hour after breakfast and one bag half an hour after dinner for 6 continuous weeks. The components of the placebo are dextrin and caramel fabricated based on the color of XPYS-HEG.

### 2.9. The Quality Control for XPYS-HEG

The XPYS-HEG is provided by the Beijing Kangren Tang Pharmaceutical Co., Ltd. The production procedure is as follows: selection of the genuine regional drug, implementation of modern pharmaceutical technology, the use of Chinese herbal fragments as raw materials according to traditional processing methods, preparing the granular formulation after single-herb extraction, concentration, and drying with decoction as a standard. The quality standards are higher than the Codex standard for enterprise internal control standards. The 95% ethanol extract of the roast milkvetch root granule is not less than 24%. Ginseng granules containing ginsenoside Rg1 (C42H72O14) and ginsenoside Re (C48H82O18) shall not be less than 0.80%, and the total ginsenoside Rb1 (C54H92O23) shall not be less than 0.18%. Tangerine peel granule contains hesperidin not below 1%. The 95% ethanol extract of nutgrass galingale rhizome processed with vinegar is not less than 10%. The ferulic acid content of the angelica formula particle is not less than 0.030%. Wolfberry polysaccharides of the barbary wolfberry fruit granule contain no less than 1.26%.

### 2.10. Evaluation

To evaluate the quality of life, we used the Chinese edition of the universal quality-of-life scale for the SF-36 (The Short Form-36 Health Survey), which was developed by the American Medical Outcomes Study Group. The SF-36 includes eight factors: physical functioning, role physical, bodily pain, general health, vitality, social functioning, role emotional, and mental health. Higher scores correspond to a better quality of life.

The measures were assessed at the baseline, at the end of intervention at the 6th week, and at the posttreatment followups conducted at the end of the 12th and 18th weeks. We compared the scores for the eight factors of the SF-36 between baseline and the end of 6th week and among the end of 6th, 12th, and 18th weeks to assess the intervention efficacy and long-term intervention effect on the quality of life of XPYS-HEG in treatment of people with FPSH and liver-qi stagnation and spleen-qi deficiency syndrome in TCM.

### 2.11. Data Entry and Statistical Analysis

A database was built by Epidata 3.0, and the data were entered twice by two different people. 

According to the principle of intention-to-treat, two data sets were used to test the difference between the drug and the placebo: full analysis sets (FAS), including all 200 participants, and per-protocol sets (PPS), including 197 participants (excluding 3 cases due to vomiting after drinking, suffering from intestinal adhesion, and catching cold and without following the treatment plan) [11] (see flow chart of the randomized controlled trial in Supplementary Materials, Supplementary Materials will be available online at doi: 10.1155/2012/509705).

The mean and standard deviation are presented for the quantitative data, and the frequency and the percentage are presented for the numerical data. A *P* value of 0.05 was considered significant. A Student's *t*-test or chi-squared test was used to test the differences among the characteristics of demography and the baseline of the SF-36 between the two groups. To detect changes in the SF-36, repeated-measures analysis of variance (ANOVA) models were used. All analyses were performed using the Statistical Package for the Social Sciences (SPSS for Windows version 17.00 SPSS Inc., Chicago, Ill).

### 2.12. Quality Control

#### 2.12.1. The Training before Clinical Trials

The researchers had a full understanding of the clinical trial scheme and measures, and they proceeded according to the outlined scheme. The researchers observed adverse events or unexpected side effects and followed up in these cases.

#### 2.12.2. Compliance

Compliance was established by explaining the trial to the participants and obtaining informed consent, interviewing the participants once every two weeks, verifying that the drug was taken, issuing the drug in the amount required for two weeks, and keeping records of the drugs issued to the participants.

## 3. Results

### 3.1. Characteristics of Demography and Baseline

There were no differences between the groups for the baseline measures of gender, ethnicity, marital status, occupation, or educational status (*P* > 0.05, see [11]) (see Table  1 in Supplementary Materials) or among the scores of the eight factors on the SF-36 (*P* > 0.05, see Table 1).

### 3.2. Comparison of the Intervention Effect between Groups

At the end of the 6th week, the analyses were conducted for the FAS and PPS with the objective data. A repeated-measures ANOVA examining the score changes of bodily pain according to the SF-36 from baseline to 6 weeks showed a significant treatment × time interaction (FAS: *P* = 0.007, PPS: *P* = 0.005) (see Table 2), changes in the score for role physical showed a significant treatment effect (FAS: *P* = 0.032, PPS: *P* = 0.026), and all eight factors showed significant time effects (*P* < 0.01).

### 3.3. Comparison of the Long-Term Clinical Effect between the Groups

Changes in the scores for the eight factors of the SF-36 at the end of the 6th, 12th, and 18th weeks were examined using repeated-measures ANOVA for the FAS and PPS. There was a significant treatment × time interaction for mental health (FAS: *P* = 0.017, PPS: *P* = 0.025) (see Table 3). Furthermore, there were significant time effects (*P* < 0.05), but no treatment × time interaction, and the other factors showed significant time effects (*P* < 0.01) with the exception of social function. There were significant treatment effects detected for five factors (*P* < 0.05), excluding physical functioning, bodily pain, and mental health.

## 4. Discussion

Quality of life includes various domains, such as physical functioning, mental status, social association, and bodily feeling. With the changes in the medical model, patient-reported outcomes (PRO), such as quality of life, are increasingly used as outcome assessments. Quality of life is regarded as an important PRO for people with FPSH and could also be used as an outcome measure to examine the effectiveness of therapies for people with FPSH [10].

**Figure 1:**
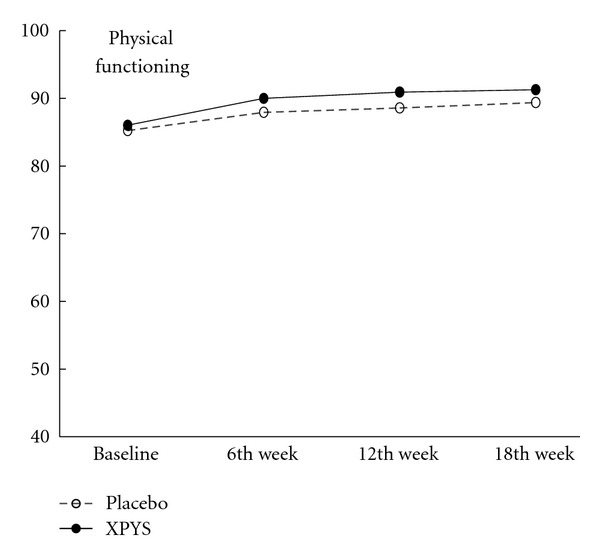
The trends of the changes in the scores for physical functioning (PPS).

**Figure 2:**
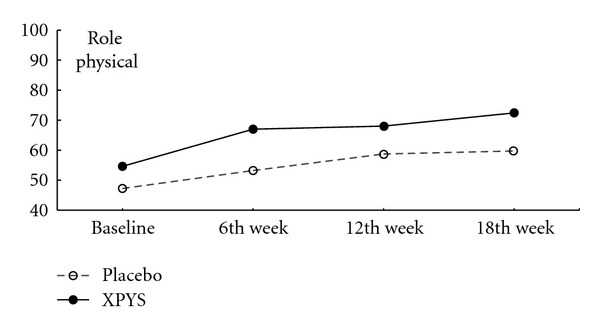
The trend of the changes in the scores for role physical (PPS).

**Figure 3:**
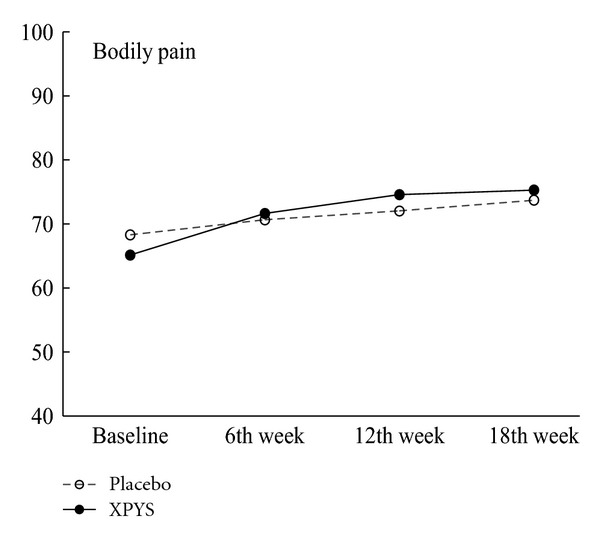
The trend in the change in scores for bodily pain (PPS).

**Figure 4:**
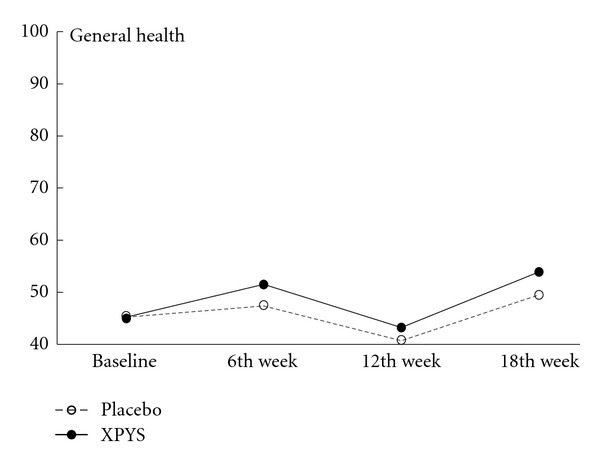
The trend in the change in scores for general health (PPS).

**Figure 5:**
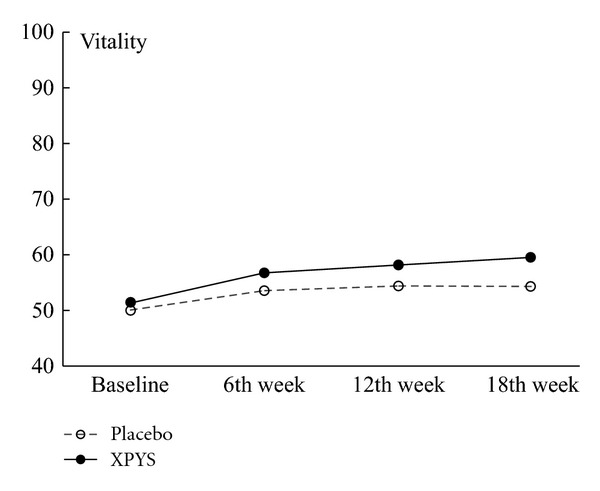
The trend for the change in scores for vitality (PPS).

**Figure 6:**
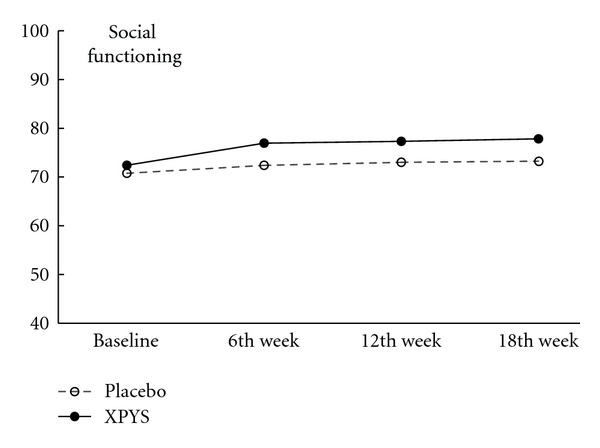
The trend for the change in scores for social function (PPS).

**Figure 7:**
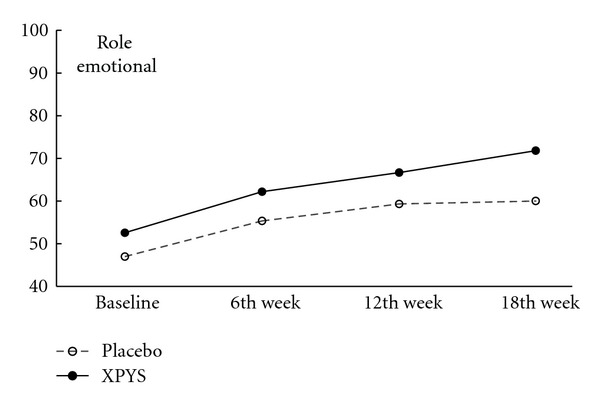
The trend for the change in scores for role emotional (PPS).

**Figure 8:**
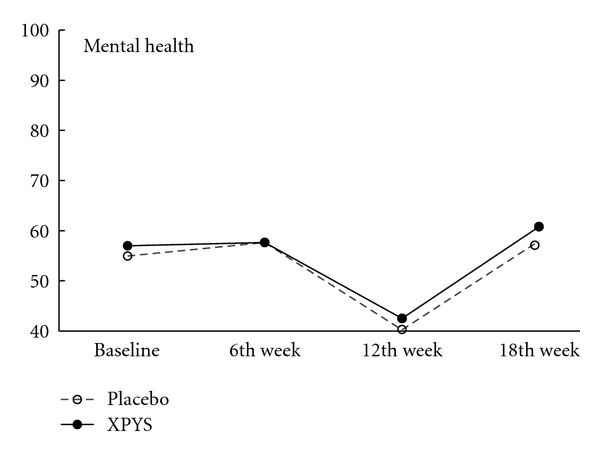
The trend for the change in scores for mental health (PPS).

The results of this study demonstrated that the scores for eight factors on the SF-36 changed statistically from baseline to the end of the 6th week of treatment in the two groups, and the changes exhibited an increasing trend (see Table 1 and Figures 1, 2, 3, 4, 5, 6, 7, and 8). Our findings suggest that the quality of life of the participants could be improved as a result of the intervention. When comparing the scores of the eight factors at the end of the 6th week between the two groups, we found that the score for the factor of role physical in the XPYS group was significantly higher than that in the placebo group. However, there was no obvious score change for the factor of role physical in the two groups according to the treatment × time interaction. Compared with the baseline score, the score change for bodily pain in the XPYS group was more obvious than in the placebo group according to the treatment × time interaction at the end of the 6th week. However, there was no obvious score change in the two groups from the end of the 6th week to the end of the 12th and 18th weeks. This result suggested that XPYS-HEG could ease bodily pain and influence the effects on work and housework that result from bodily pain in people with FPSH and liver-qi stagnation and spleen-qi deficiency syndrome. However, there was no a long-term effect.

The scores for seven of the factors on the SF-36 changed significantly between the two groups during the follow-up period, with the exception of social functioning from the end of the 6th week to the end of the 12th and 18th weeks. This finding indicated that there was a long-term effect of at least 18 weeks on quality of life in the two groups. The scores for the factors of general health and mental health are also worth noting. Compared with the baseline scores and the scores at the end of the 6th week, the scores of these two factors decreased at the end of the 12th week, but they increased at the end of the 18th week. The two factors that were related to the level of recognition of health among the participants were influenced by the varying conditions. When comparing the scores of the eight factors at the end of the 18th week between the two groups, we found that the scores for the factors of role physical, general health, vitality, social functioning, and role emotional in the XPYS group were significantly higher than those in the placebo group. However, there were no obvious changes in the scores for these factors between the two groups based on the comparison of the treatment × time interaction. Compared with the scores at the end of 6th week, the change in the score for mental health in the XPYS group was more obvious than that in the placebo group according to the treatment × time interaction at the end of the 12th and 18th weeks. However, there was no obvious change in the scores in the two groups from baseline to the end of the 6th week. This result suggested that XPYS-HEG had a slow-acting effect that could stimulate a positive mood and ease a negative mood. 

XPYS-HEG is composed by Radix Astragali, Radix Ginseng, Pericarpium Citri Reticulatae, Rhizoma Cyperi, Radix Angelicae, and Fructus Lycii. Radix Astragali and Radix Ginseng are used to replenish spleen qi; Pericarpium Citri Reticulatae and Rhizoma Cyperi are applied to soothe liver qi; Radix Angelicae and Fructus Lycii nourish the blood. According to the theory of TCM, the effect of XPYS-HEG on the quality of life in participants is explained as follows. (1) The spleen governs the muscles, flesh and the four limbs. Replenishing the spleen qi and nourishing the blood can nourish the four limbs. The liver governs the sinews, and when the liver obtains blood, the sinews stretch, which may explain why XPYS-HEG could ease both bodily pain and influence the work and housework resulting from bodily pain among people with FPSH and liver-qi stagnation and spleen-qi deficiency syndrome. (2) Dispersing and discharging functions of the liver can regulate emotion. The regulation of emotion requires a long period, which may explain why XPYS-HEG was observed to stimulate a positive mood and ease a negative mood at the end of the 18th week during the posttreatment follow-up period.

Additionally, according to Figures 5 and 7, there were changes in the trends for the scores of the factors for vitality and role emotional, which increased gradually in the XPYS group, but decreased gradually or were maintained in the placebo group. Further research is required to identify the long-term effect of XPYS-HEG for improving vitality and easing functional constraints resulting from a negative mood.

## 5. Conclusions 

XPYS-HEG could partially improve the quality of life for people with FPSH and liver-qi stagnation and spleen-qi deficiency syndrome. XPYS-HEG may ease bodily pain, stimulate a positive mood and ease a negative mood.

## Supplementary Material

There were no differences between groups for baseline measures of gender, ethnicity, marital status, occupations and educational status.
Two data sets were used to test the difference between the drug and the placebo in this study. Full analysis sets (FAS), including all 200 participants, and per-protocol sets (PPS), including 197 participants (excluding 3 cases due to vomiting after drinking, suffering from intestinal adhesion, and catching cold and without following the treatment plan).

## Figures and Tables

**Table 1:** Baseline comparison of factor scores for the SF-36 between the two groups $\left(\overset{-}{\chi }\pm \;s\right)$.

		Placebo	XPYS	*Z* value	*P* value
Physical functioning	FAS	85.25 ± 11.53	85.70 ± 12.79	−0.509	0.610
	PPS	85.25 ± 11.53	86.03 ± 12.52	−0.644	0.520
Role physical	FAS	47.25 ± 36.39	54.25 ± 35.90	−1.376	0.169
	PPS	47.25 ± 36.39	54.64 ± 36.14	−1.434	0.152
Bodily pain	FAS	68.29 ± 14.59	65.54 ± 13.12	−1.513	0.130
	PPS	68.29 ± 14.59	65.13 ± 12.83	−1.643	0.100
General health	FAS	45.69 ± 13.66	45.68 ± 16.19	−0.158	0.874
	PPS	45.69 ± 13.66	45.24 ± 16.03	−0.345	0.730
Vitality	FAS	50.05 ± 13.46	51.60 ± 15.17	−0.661	0.538
	PPS	50.05 ± 13.46	51.29 ± 15.14	−0.457	0.648
Social functioning	FAS	70.75 ± 17.70	72.13 ± 16.17	−0.511	0.610
	PPS	70.75 ± 17.70	72.42 ± 16.23	−0.652	0.514
Role emotional	FAS	47.00 ± 35.17	52.33 ± 33.59	−1.079	0.281
	PPS	47.00 ± 35.17	52.58 ± 33.62	−1.113	0.266
Mental health	FAS	54.96 ± 14.54	56.76 ± 13.41	−0.838	0.402
	PPS	54.96 ± 14.54	56.99 ± 13.38	−0.942	0.346

**Table 2:** The comparison of factor scores from the SF-36 from baseline to the end of the 6th week between the two groups $\left(\overset{-}{\chi }\pm \;s\right)$.

		Group	Baseline	6th week	Time effect		Treatment × time interaction		Treatment effect	
					*F*	*P*	*F*	*P*	*F*	*P*
Physical functioning	FAS	Placebo	85.25 ± 11.53	87.95 ± 10.28	23.501	0.000^∗^	0.777	0.379	0.585	0.445
		XPYS	85.70 ± 12.79	89.60 ± 8.22						
	PPS	Placebo	85.25 ± 11.53	87.95 ± 10.28	23.313	0.000^∗^	0.844	0.359	1.110	0.293
		XPYS	86.03 ± 12.52	90.00 ± 7.32						

Role physical	FAS	Placebo	47.25 ± 36.39	53.25 ± 36.18	24.286	0.000^∗^	2.848	0.093	4.686	0.032^∗^
		XPYS	54.25 ± 35.90	66.50 ± 33.75						
	PPS	Placebo	47.25 ± 36.39	53.25 ± 36.18	23.950	0.000^∗^	2.880	0.091	5.033	0.026^∗^
		XPYS	54.64 ± 36.14	67.01 ± 33.57						

Bodily pain	FAS	Placebo	68.29 ± 14.59	70.65 ± 14.03	36.080	0.000^∗^	7.468	0.007^∗^	0.195	0.660
		XPYS	65.54 ± 13.12	71.84 ± 12.16						
	PPS	Placebo	68.29 ± 14.59	70.65 ± 14.03	36.804	0.000^∗^	8.025	0.005^∗^	0.380	0.538
		XPYS	65.13 ± 12.83	71.63 ± 11.98						

General health	FAS	Placebo	45.69 ± 13.66	47.59 ± 13.71	7.929	0.005^∗^	2.146	0.145	2.006	0.158
		XPYS	45.68 ± 16.19	51.70 ± 13.34						
	PPS	Placebo	45.69 ± 13.66	47.59 ± 13.71	8.249	0.005^∗^	2.370	0.125	1.475	0.226
		XPYS	45.24 ± 16.03	51.53 ± 13.21						

Vitality	FAS	Placebo	50.05 ± 13.46	53.55 ± 14.71	27.812	0.000^∗^	1.164	0.282	1.671	0.198
		XPYS	51.60 ± 15.17	56.90 ± 15.17						
	PPS	Placebo	50.05 ± 13.46	53.55 ± 14.71	28.076	0.000^∗^	1.348	0.247	1.355	0.246
		XPYS	51.29 ± 15.14	56.75 ± 15.23						

Social functioning	FAS	Placebo	70.75 ± 17.70	72.38 ± 15.82	10.143	0.002^∗^	2.235	0.137	1.867	0.173
		XPYS	72.13 ± 16.17	76.63 ± 14.40						
	PPS	Placebo	70.75 ± 17.70	72.38 ± 15.82	9.898	0.002^∗^	2.189	0.141	2.264	0.134
		XPYS	72.42 ± 16.23	76.93 ± 14.25						

Role emotional	FAS	Placebo	47.00 ± 35.17	55.33 ± 33.58	21.020	0.000^∗^	0.115	0.735	2.002	0.159
		XPYS	52.33 ± 33.59	62.00 ± 29.60						
	PPS	Placebo	47.00 ± 35.17	55.33 ± 33.58	20.385	0.000^∗^	0.105	0.746	2.136	0.146
		XPYS	52.58 ± 33.62	62.20 ± 29.12						

Mental health	FAS	Placebo	54.96 ± 14.54	57.64 ± 14.64	8.009	0.005^∗^	3.024	0.084	0.172	0.679
		XPYS	56.76 ± 13.41	57.40 ± 13.04						
	PPS	Placebo	54.96 ± 14.54	57.64 ± 14.64	7.861	0.006^∗^	2.876	0.091	0.291	0.590
		XPYS	56.99 ± 13.38	57.65 ± 12.98						

**Table 3:** The comparison of factor scores from the SF-36 from the end of the 6th week to the end of the 12th and 18th weeks between the two groups $\left(\overset{-}{\chi }\pm \;s\right)$.

			6th week	12th week	18th week	Time effect		Treatment × time interaction		Treatment effect	
						*F*	*P*	*F*	*P*	*F*	*P*
Physical functioning	FAS	Placebo	87.95 ± 10.28	88.60 ± 9.40	89.40 ± 8.36	5.992	0.003^∗^	0.306	0.737	1.943	0.165
		XPYS	89.60 ± 8.22	90.50 ± 8.03	90.85 ± 8.91						
	PPS	Placebo	87.95 ± 10.28	88.60 ± 9.40	89.40 ± 8.36	5.996	0.003^∗^	0.305	0.738	3.336	0.069
		XPYS	90.00 ± 7.32	90.93 ± 7.05	91.29 ± 8.05						

Role physical	FAS	Placebo	53.25 ± 36.18	58.75 ± 34.88	59.75 ± 35.34	7.273	0.001^∗^	1.471	0.232	6.347	0.013^∗^
		XPYS	66.50 ± 33.75	67.50 ± 32.08	71.75 ± 30.91						
	PPS	Placebo	53.25 ± 36.18	58.75 ± 34.88	59.75 ± 35.34	7.252	0.001^∗^	1.445	0.238	6.963	0.009^∗^
		XPYS	67.01 ± 33.57	68.04 ± 31.82	72.42 ± 30.51						

Bodily pain	FAS	Placebo	70.65 ± 14.03	72.04 ± 13.24	73.71 ± 12.72	15.083	0.000^∗^	1.223	0.297	1.242	0.266
		XPYS	71.84 ± 12.16	74.70 ± 11.97	75.38 ± 10.71						
	PPS	Placebo	70.65 ± 14.03	72.04 ± 13.24	73.71 ± 12.72	15.194	0.000^∗^	1.293	0.277	1.053	0.306
		XPYS	71.63 ± 11.98	74.58 ± 11.82	75.28 ± 10.51						

General health	FAS	Placebo	47.59 ± 13.71	40.71 ± 9.73	49.53 ± 12.90	140.896	0.000^∗^	0.948	0.389	5.783	0.017^∗^
		XPYS	51.70 ± 13.34	43.56 ± 9.94	53.95 ± 13.07						
	PPS	Placebo	47.59 ± 13.71	40.71 ± 9.73	49.53 ± 12.90	142.937	0.000^∗^	1.266	0.284	5.340	0.022^∗^
		XPYS	51.53 ± 13.21	43.26 ± 9.33	53.92 ± 12.89						

Vitality	FAS	Placebo	53.55 ± 14.71	54.40 ± 14.55	54.30 ± 14.74	4.233	0.016^∗^	1.527	0.220	4.525	0.035^∗^
		XPYS	56.90 ± 15.17	58.25 ± 14.13	59.60 ± 13.48						
	PPS	Placebo	53.55 ± 14.71	54.40 ± 14.55	54.30 ± 14.74	4.302	0.015^∗^	1.592	0.206	4.225	0.041^∗^
		XPYS	56.75 ± 15.23	58.14 ± 14.17	59.54 ± 13.50						

Social functioning	FAS	Placebo	72.38 ± 15.82	73.00 ± 15.76	73.25 ± 16.19	0.807	0.447	0.025	0.975	4.432	0.037^∗^
		XPYS	76.63 ± 14.40	77.00 ± 13.96	77.50 ± 13.76						
	PPS	Placebo	72.38 ± 15.82	73.00 ± 15.76	73.25 ± 16.19	0.807	0.448	0.025	0.975	5.126	0.025^∗^
		XPYS	76.93 ± 14.25	77.32 ± 13.78	77.84 ± 13.56						

Role emotional	FAS	Placebo	55.33 ± 33.58	59.33 ± 34.36	60.00 ± 34.49	9.058	0.000^∗^	1.524	0.220	4.152	0.043^∗^
		XPYS	62.00 ± 29.60	66.33 ± 27.01	71.33 ± 28.04						
	PPS	Placebo	55.33 ± 33.58	59.33 ± 34.36	60.00 ± 34.49	9.169	0.000^∗^	1.617	0.201	4.514	0.035^∗^
		XPYS	62.20 ± 29.12	66.67 ± 26.35	71.82 ± 27.36						

Mental health	FAS	Placebo	57.64 ± 14.64	40.24 ± 11.11	57.96 ± 13.42	1045.885	0.000^∗^	4.148	0.017^∗^	0.782	0.378
		XPYS	57.40 ± 13.04	42.52 ± 11.08	60.48 ± 13.20						
	PPS	Placebo	57.64 ± 14.64	40.24 ± 11.11	57.96 ± 13.42	1152.427	0.000^∗^	3.767	0.025^∗^	0.983	0.323
		XPYS	57.65 ± 12.98	42.52 ± 11.11	60.83 ± 13.08						
