# Supplementary material for: Xiaopiyishen Herbal Extract Granule Improves the Quality of Life among People with Fatigue-Predominant Subhealth and Liver-Qi Stagnation and Spleen-Qi Deficiency Syndrome
Source: Evid Based Complement Alternat Med. 2012 Jul 18;2012:509705. doi: 10.1155/2012/509705 (PMC3407644; doi:10.1155/2012/509705)
Supplement: Supplementary file 1 — There were no differences between groups for baseline measures of gender, ethnicity, marital status, occupations and educational status. Two data sets were used to test the difference between the drug and the placebo in this study. Full analysis sets (FAS), including all 200 participants, and per-protocol sets (PPS), including 197 participants (excluding 3 cases due to vomiting after drinking, suffering from intestinal adhesion, and catching cold and without following the treatment plan). [file 509705.f1.doc]

**Table1 Baseline demographics Comparison Between the Two Groups**

| Items | Characteristic | Data　sets | Placebo N（%） | XPYS N（%） | *x^2^* | *P* |
| --- | --- | --- | --- | --- | --- | --- |
| Gender | male | FAS | 29（29.00） | 33（33.00） | 0.374 | 0.541 |
|  | female |  | 71（71.00） | 67（67.00） |  |  |
|  | male | PPS | 29（29.00） | 31（31.96） | 0.204 | 0.652 |
|  | female |  | 71（71.00） | 66（68.04） |  |  |
| Ethnicity* | The Han nationality | FAS | 96（96.00） | 96（96.00） | - | 0.683 |
|  | Huis |  | 4（4.00） | 2（2.00） |  |  |
|  | Manchus |  | 0（0） | 1（1.00） |  |  |
|  | Mongolian |  | 0（0） | 1（1.00） |  |  |
|  | The Han nationality | PPS | 96（96.00） | 93（95.88） | - | 0.5 |
|  | Huis |  | 4（4.00） | 2（2.06） |  |  |
|  | Manchus |  | 0（0.00） | 1（1.03） |  |  |
|  | Mongolian |  | 0（0.00） | 1（1.03） |  |  |
| Marital Status* | Unmarried | FAS | 16（16.00） | 15（15.00） | - | 0.712 |
|  | Married |  | 81（81.00） | 82（82.00） |  |  |
|  | Widowed |  | 1（1.00） | 2（2.00） |  |  |
|  | Separated |  | 0（0.00） | 1（1.00） |  |  |
|  | Divorced |  | 2（2.00） | 0（0） |  |  |
|  | Unmarried | PPS | 16（16.00） | 14（14.43） | - | 0.665 |
|  | Married |  | 81（81.00） | 80（82.47） |  |  |
|  | Widowed |  | 1（1.00） | 2（2.06） |  |  |
|  | Separated |  | 0（0.00） | 1（1.03） |  |  |
|  | Divorced |  | 2（2.00） | 0（0.00） |  |  |
| Occupation* | Workman | FAS | 4（4.00） | 1（1.00） | - | 0.217 |
|  | Peasant |  | 1（1.00） | 0（0） |  |  |
|  | Manager |  | 17（17.00） | 26（26.00） |  |  |
|  | Teaching staff |  | 12（12.00） | 14（14.00） |  |  |
|  | Medical satff |  | 30（30.00） | 33（33.00） |  |  |
|  | Researcher |  | 1（1.00） | 2（2.00） |  |  |
|  | Serviceman |  | 2（2.00） | 3（3.00） |  |  |
|  | Self-employed staff |  | 7（7.00） | 5（5.00） |  |  |
|  | Office worker |  | 22（22.00） | 9（9.00） |  |  |
|  | Retiree |  | 2（2.00） | 2（2.00） |  |  |
|  | Other |  | 2（2.00） | 5（5.00） |  |  |
|  | Workman | PPS | 4（4.00） | 0（0.00） | - | 0.131 |
|  | Peasant |  | 1（1.00） | 0（0.00） |  |  |
|  | Manager |  | 17（17.00） | 25（25.77） |  |  |
|  | Teaching staff |  | 12（12.00） | 13（13.40） |  |  |
|  | Medical satff |  | 30（30.00） | 33（34.02） |  |  |
|  | Researcher |  | 1（1.00） | 2（2.06） |  |  |
|  | Serviceman |  | 2（2.00） | 3（3.09） |  |  |
|  | Self-employed staff |  | 7（7.00） | 5（5.15） |  |  |
|  | Office worker |  | 22（22.00） | 9（9.28） |  |  |
|  | Retiree |  | 2（2.00） | 2（2.06） |  |  |
|  | Other |  | 2（2.00） | 5（5.15） |  |  |
| Educational status* | Junior high school | FAS | 1（1.00） | 3（3.00） | - | 0.262 |
|  | Senior high school and technical secondary school |  | 22（22.00） | 12（12.00） |  |  |
|  | Junior college and college |  | 63（63.00） | 72（72.00） |  |  |
|  | Graduate |  | 14（14.00） | 13（13.00） |  |  |
|  | Junior high school | PPS | 1（1.00） | 3（3.09） | - | 0.262 |
|  | Senior high school and technical secondary school |  | 22（22.00） | 12（12.37） |  |  |
|  | Junior college and college |  | 63（63.00） | 69（71.13） |  |  |
|  | Graduate |  | 14（14.00） | 13（13.40） |  |  |

*: using the method of fisher’s exact probability test.

There were no differences between groups for baseline measures of gender, ethnicity, marital status, occupations and educational status.

**
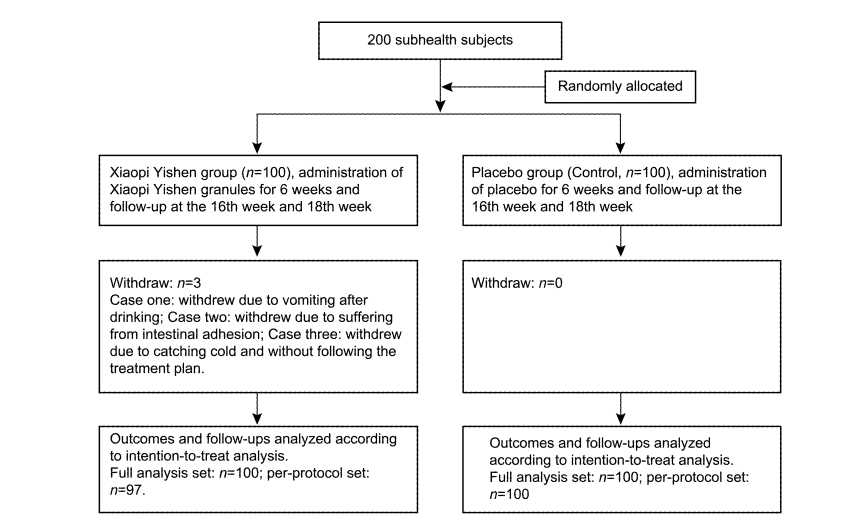
**

**Figure Flow Chart of the Randomized Controlled Trial**

Two data sets were used to test the difference between the drug and the placebo in this study. Full analysis sets (FAS), including all 200 participants, and per-protocol sets (PPS), including 197 participants (excluding 3 cases due to vomiting after drinking, suffering from intestinal adhesion, and catching cold and without following the treatment plan).
